# Unexpected Percutaneous Ventricular Assist Device Failure in a Complex Case of Infarct-Related Cardiogenic Shock

**DOI:** 10.1016/j.jaccas.2025.103538

**Published:** 2025-06-04

**Authors:** Sarah Louise Duus Holle, Jacob Eifer Møller, Hans-Henrik Tilsted, Helle Søholm

**Affiliations:** a Department of Cardiology, The Heart Center, Copenhagen University Hospital, Rigshospitalet, Copenhagen, Denmark; b Department of Cardiology, Odense University Hospital, Odense, Denmark; c Department of Cardiology, Skånes University Hospital, Lund, Sweden

**Keywords:** acute coronary syndrome, acute heart failure, cardiac assist devices, hemodynamics, myocardial infarction, percutaneous coronary intervention

## Abstract

**Background:**

Infarct-related cardiogenic shock (CS) is a serious complication. The implementation of the Society for Cardiovascular Angiography and Interventions (SCAI) shock classification system facilitates the timely identification and intervention in CS.

**Case Summary:**

A patient presenting with anterior ST-segment elevation myocardial infarction arrived at the catheterization laboratory in SCAI stage B, with rapid progression to stage E. Acute bedside echocardiography revealed an ejection fraction of 10%. The patient was stabilized with norepinephrine and a microaxial flow pump. The device malfunctioned, resulting in replacement.

**Discussion:**

SCAI stages need frequent reassessment to guide appropriate interventions. The microaxial flow pump was selected owing to its less invasive nature and its ability to provide left ventricular support. The decision to implant a mechanical circulatory support device is complex and must be made swiftly in response to the patient’s clinical condition.

## Graphical Abstract

**Figure:**
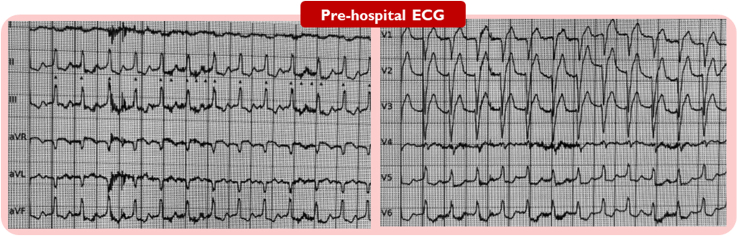

## History of Presentation

**Figure 1:**
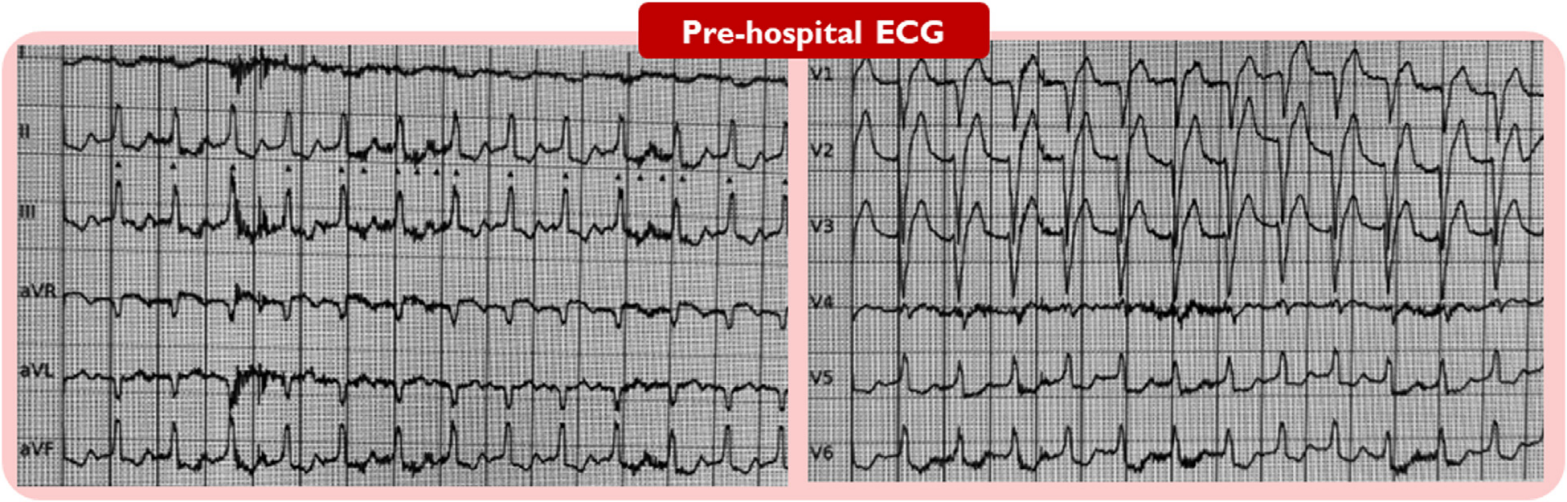
Electrocardiography Obtained Before Arrival at Hospital

**Figure 2:**
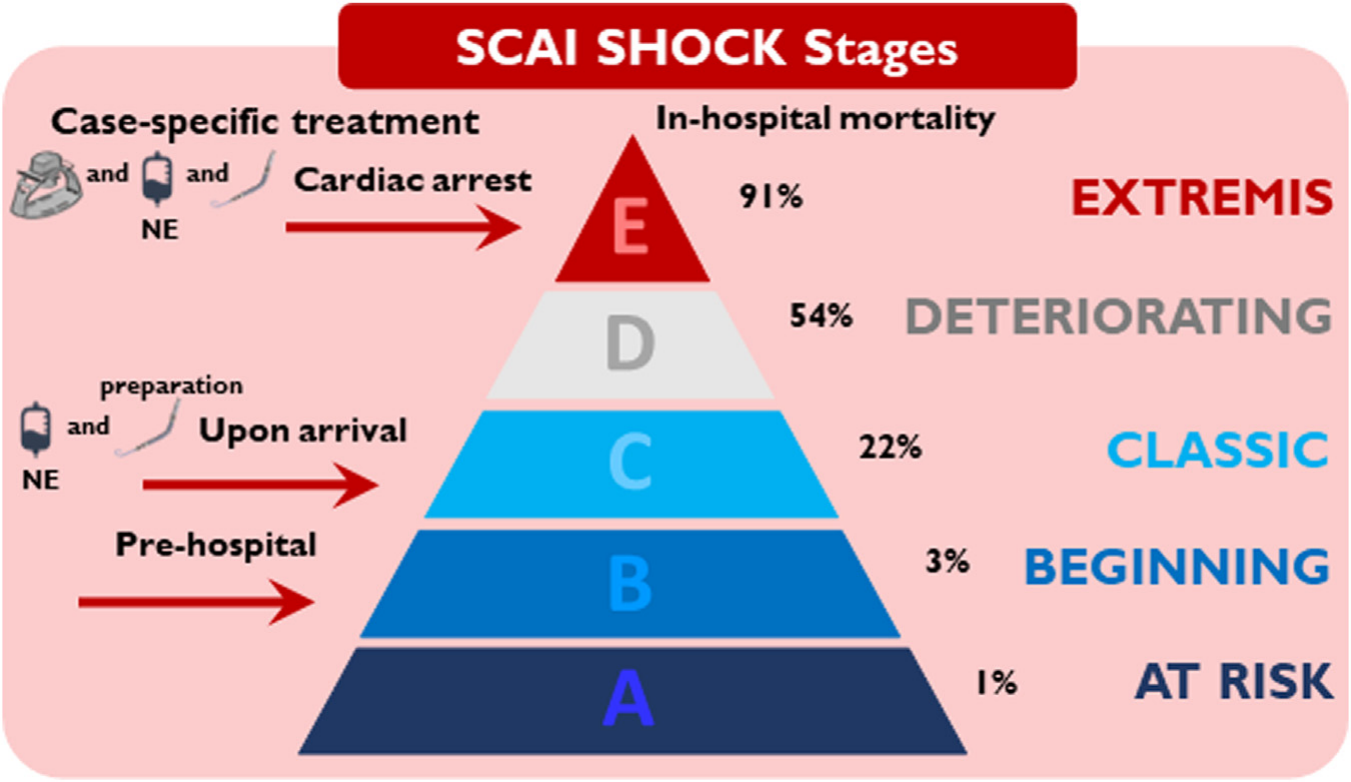
SCAI SHOCK Stages Including Pharmacologic and Mechanical Support NE = norepinephrine.

A 69-year-old man experienced acute onset of chest pain at midnight accompanied by near-syncope, but did not seek medical attention until the following morning. The pre-hospital ambulance electrocardiogram demonstrated anterior ST-segment elevations with tachycardia, with a frequency of up to 130 beats/min) (Figure 1). He was directly transferred for acute coronary angiography (CAG) at a tertiary heart center. On arrival, he was clammy and pale with a blood pressure of 104/72 mm Hg. An acute bedside echocardiogram revealed a left ventricular ejection fraction of approximately 10%, with no substantial right-side heart failure, evidence of mechanical complications or severe valvular disease) (Video 1). Owing to progressive hypotension and an arterial lactate level of 10.2 mmol/L, norepinephrine infusion was initiated. The shock team was gathered for a discussion of the treatment strategy. A microaxial flow pump was decided on before the CAG, because the patient fulfilled the inclusion criteria in the recent DanGer Shock trial.^1^ While the device and femoral access were being prepared, the patient experienced a short episode of cardiac arrest with pulseless electrical activity and need for chest compression. Return of spontaneous circulation was achieved. However, the patient subsequently experienced another episode of cardiac arrest, prompting the decision to intubate. According to the Society for Cardiovascular Angiography and Interventions (SCAI) shock staging system,^2^ the patient progressed rapidly from stage B in the pre-hospital setting to stage C at arrival and to stage E during preparation of the microaxial flow pump, highlighting the urgent need for mechanical circulatory support) (Figure 2). During mechanical cardiopulmonary resuscitation, a microaxial flow pump (Impella CP) was implanted through the right femoral artery (14-F sheath) started at a low “performance (P) level” and gradually increased to a flow of 3.5 L/min at P-level P8. Hemodynamic stability was immediately achieved.

**Take-Home Messages**

• Cardiogenic shock is a severe and complex complication of STEMI.
• SCAI stages of cardiogenic shock are dynamic and should be frequently reassessed based on established criteria to guide appropriate interventions.
• The choice to implant a mechanical circulatory support device is complex and should be tailored to the individual patient’s clinical circumstances.
• When a device fails to provide adequate support, a problem-focused approach including repeated echocardiography is essential. If the underlying issue cannot be identified or solved, device replacement is required.

## Past Medical History

The patient was an active smoker with no significant medical history.

## Differential Diagnosis

STEMI-related cardiogenic shock (CS) was the primary diagnosis based on ST-segment elevation and the clinical course, warranting further investigation. Stress cardiomyopathy, sepsis-induced myocardial dysfunction, and type II myocardial infarction were to be considered as differential diagnoses if the CAG revealed normal coronary arteries without significant stenosis.

## Investigations

**Figure 3:**
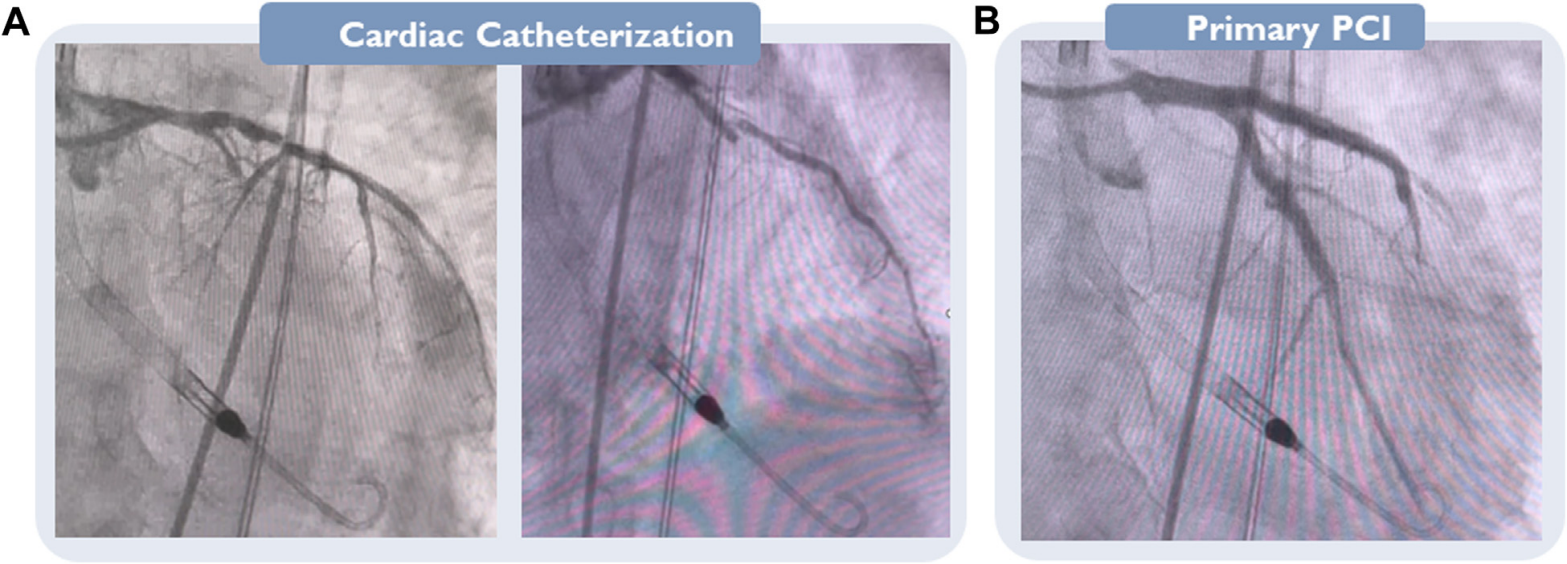
Coronary Angiography Examination andPCI (A) Coronary angiography; (B) primary percutaneous coronary intervention (PCI).

CAG was performed via transradial access (6-F sheath). The CAG showed a 70% proximal and 99% mid-segment occlusion of the left anterior descending coronary artery (LAD), 80% proximal and 100% mid-segment occlusion of the left circumflex coronary artery (LCx), and complete (100%) occlusion of the right coronary artery (RCA) (Figure 3A, Videos 2A and 2B).

## Management

Primary percutaneous coronary intervention (pPCI) of the left main coronary artery (LM) to the LAD and LCx was performed by means of the Culotte technique, placing 2 drug-eluting stents (Xience Pro 3.5 × 48 mm and 3.0 × 28 mm) with a good result (Figure 3B, Video 3). During the pPCI, the microaxial flow pump triggered a “high purge pressure” alarm. The purge tubes were checked for kinks, which were not found, and it was decided to replace both the purge fluid and the purge cassette; the problem was thus resolved, and the patient was transferred to the cardiac intensive care unit (CICU) after the pPCI. In the CICU, motor current increased on the device, leading to device malfunction with pump stop. Bedside echocardiography was performed to ensure correct position of the microaxial flow pump and to rule out presence of thrombus formation, which was not present. Several attempts to run the device were attempted, but it was only possible to run at P-level 2 with an inadequate flow and massive elevation of lactate up to 21 mmol/L. The device was therefore replaced by a new device via the same access site.

## Outcome and Follow-Up

**Figure 4:**
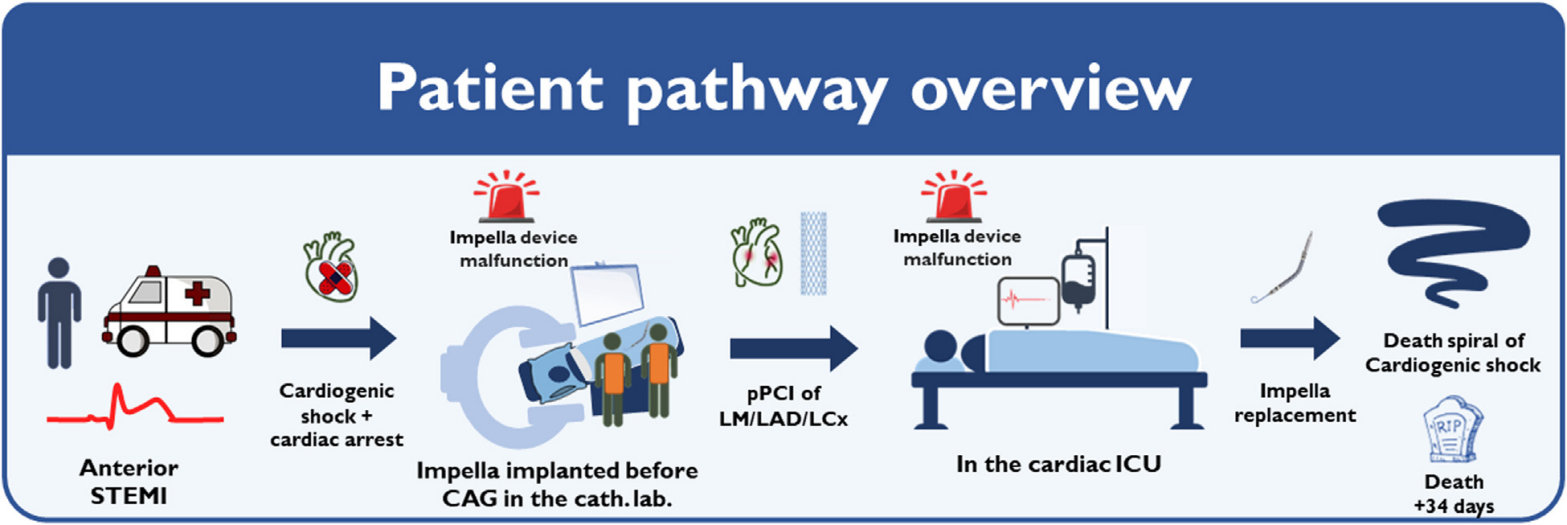
Overview of the Patient’s Pathway CAG = coronary angiography; cath. lab. = catheterization laboratory; ICU = intensive care unit; LAD = left anterior descending; LCx = left circumflex; LM = left main; pPCI = primary percutaneous coronary intervention; STEMI = ST-segment elevation myocardial infarction.

In the following weeks, the patient’s cardiac function increased to an ejection fraction of 30%. He was hemodynamically stabilized to the extent that a microaxial flow pump was no longer required. However, he experienced a prolonged complicated ICU course. His condition was complicated by the development of melena, atrial flutter, acute kidney failure, pneumonia, and ileus. His condition continued to decline owing to multiorgan failure, and he died after 34 days) (Figure 4).

## Discussion

CS is a severe complication of ST-segment elevation myocardial infarction (STEMI).^3^ Prompt recognition and appropriate management of CS are crucial to ensure optimal patient outcomes. The present case highlights the complexity of CS and addresses critical device malfunctions through a problem-solving approach.

The SCAI shock classifications allow prompt recognition and management of CS.^2^ Notably, the patient was in SCAI stage B both before and on arrival at the hospital. In the catheterization laboratory, the patient rapidly progressed from SCAI stage C to E within minutes. This case demonstrates that SCAI stages are dynamic rather than static, highlighting the importance of frequent reassessment based on established criteria to guide appropriate interventions.

Time is a critical factor; a delay in pPCI worsens the patient outcomes, including an increased risk for CS development.^3^ In this case the patient delay before contacting the health care system was more than 10 hours. Multivessel disease is common in STEMI patients, and it is even more prevalent in patients developing CS.^4^ pPCI of the culprit lesions (LM, LAD, LCx)was performed, and RCA was identified with a chronic total occlusion.^4^ The management of STEMI patients requires rapid and specialized care, especially when it is complicated by CS development. It is of utmost importance to transfer STEMI patients directly to high-volume primary PCI centers, because CS is a rare condition requiring more advanced care than just revascularization. Implantation of mechanical circulatory support devices can be life saving. However, the decision to use veno-arterial extracorporeal membrane oxygenation (VA-ECMO) or a microaxial flow pump is inherently difficult and must be made quickly owing to the patient’s acute and critical condition. Treatment strategies must be tailored on an individual basis and should be guided by clinical circumstances, patient characteristics, including age, and institutional resources. Age is an important consideration in selecting the most appropriate device for patient management.^5^ When VA-ECMO is being considered, patients should, if possible, be assessed for eligibility for long-term mechanical circulatory support (eg, HeartMate) or heart transplantation. VA-ECMO provides prolonged comprehensive cardiac and respiratory support. ^6^ On the other hand, a microaxial flow pump is designed for short-term mechanical support of the left ventricle. The latest microaxial flow pump can provide support for up to 30 days. The advantages of a microaxial flow pump include that it is less invasive and is associated with lower risk of complications compared with VA-ECMO.^7^ In selected patients, a microaxial flow pump can enhance coronary perfusion, thereby improving outcomes in those with infarct-related CS.^1^

In this case, a microaxial flow pump was chosen as the mechanical circulatory support device. This decision was guided by the invasive cardiologist and the shock team’s assessment of the patient before cardiac arrest. Notably, this patient met the eligibility criteria for inclusion in the DanGer Shock trial at the time of presentation.^1^ Proceeding with this microaxial flow pump–based strategy after the patient experienced cardiac arrest resulted in a significantly shorter time to circulatory support compared with activating the VA-ECMO team. Our clinical experience with initiating support with the use of a microaxial flow pump during in-hospital cardiac arrest has been positive.^8^ The present patient initially demonstrated good circulatory recovery with the microaxial flow pump, and escalation to VA-ECMO in the catheterization lab was therefore deemed unnecessary. Given the patient’s subsequent hemodynamic improvement and lactate clearance over the following 12-24 hours, no escalation to ECMELLA was performed. Whether support from VA-ECMO could have provided a better outcome remains speculative.

**Figure 5:**
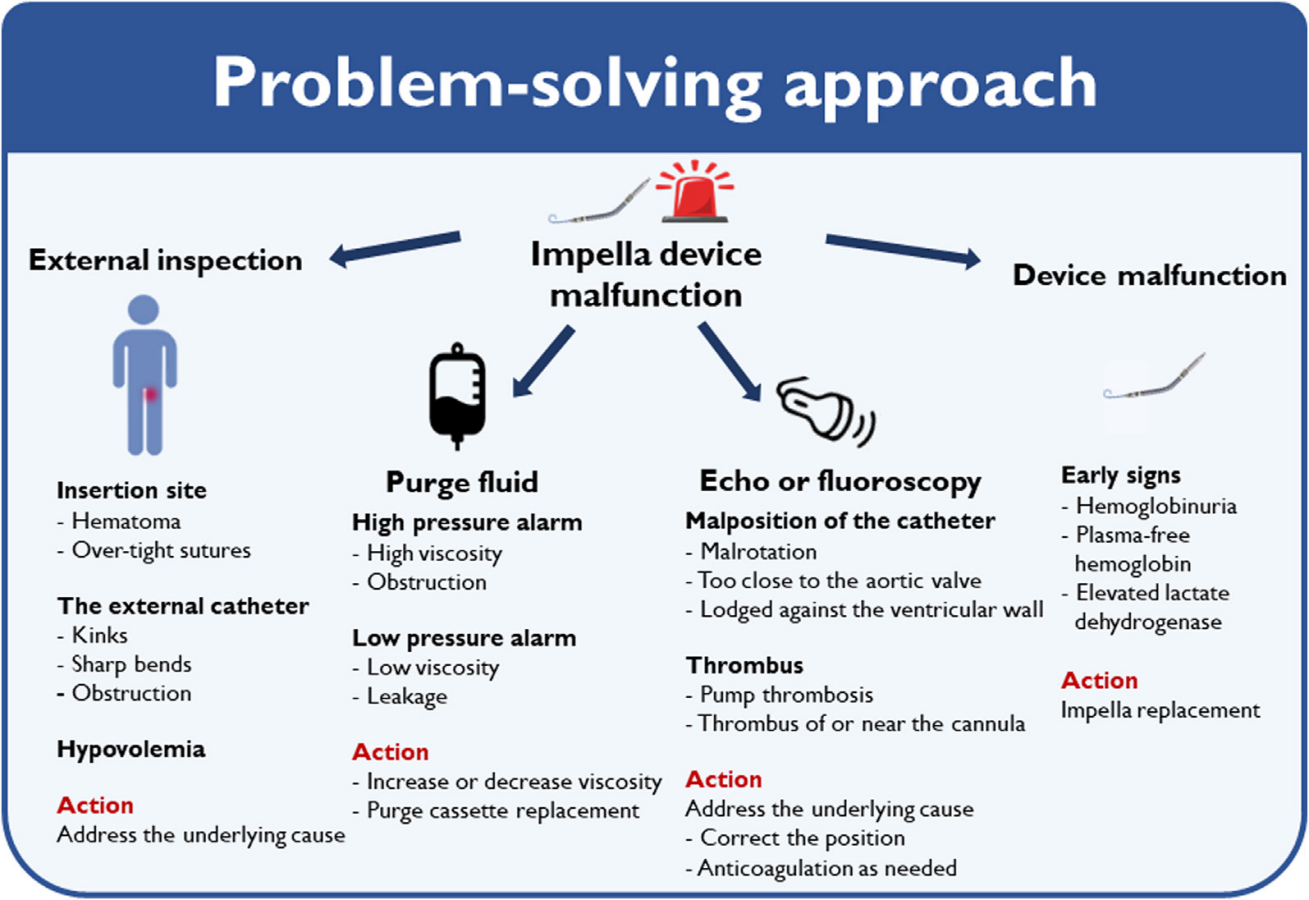
Problem-Solving Approach of Managing Impella Device Malfunction

For this patient, the treatment with mechanical support was complicated by a device malfunction of the microaxial flow pump first during the CAG and then in the CICU. On hospital arrival, the patient was in CS and quickly progressed to cardiac arrest, with persistent hemodynamic instability. He depended on the microaxial flow pump to ensure sufficient cardiac output. When the device failed to provide the necessary support, it was crucial to adopt a problem-focused approach to identify the cause of the failure (Figure 5). The problem of high motor current will develop in cases of ingestion of thrombus in the catheter, when the device will stop to avoid embolization of thrombus. No thrombus was seen either before or during troubleshooting, and the activated clotting time was within normal limits. Thus, the high motor current was thought to be caused by mechanical malfunction, with no solution other than replacing the device. In other cases, it is advisable to investigate signs of hemolysis as early indications of device malfunction. However, in this case the microaxial flow pump malfunctioned shortly after implantation, so hemolysis parameters, such as hemoglobinuria, elevated LDH, and plasma-free hemoglobin, were not available. This case represents a rare occurrence of a microaxial flow pump malfunction.

## Conclusions

CS is a severe and complex complication of STEMI, requiring dynamic reassessment through SCAI stages to guide timely interventions. The selection of mechanical circulatory support device should be individualized based on the patient’s clinical condition. In cases where a device fails to provide sufficient support, a targeted problem-solving approach is crucial, with device replacement being necessary if the underlying issue cannot be solved.

## Funding Support and Author Disclosures

Dr Møller has received Institutional research grants from Abiomed Johnson & Johnson and Novo Nordisk Foundation. All other authors have no relationships relevant to the contents of this paper to disclose.

## Appendix

Video 1 Acute bedside echocardiography in the catheterization laboratory.

Video 2A Coronary angiography examination.

Video 2B Coronary angiography examination.

Video 3 After primary percutaneous coronary intervention.
